# Inflammation as a Key Outcome Pathway in Particle Induced Effects in the Lung

**DOI:** 10.3389/fpubh.2022.869041

**Published:** 2022-05-25

**Authors:** Paul J. A. Borm, Dominique Lison, Kevin Driscoll, Rodger Duffin, Jack Harkema, Klaus Weber, Alison Elder

**Affiliations:** ^1^Heinrich Heine University of Dusseldorf, Dusseldorf, Germany; ^2^Nanoconsult Holding BV, Meerssen, Netherlands; ^3^Louvain Centre for Toxicology and Applied Pharmacology, Universite Catholique Louvain, Brussels, Belgium; ^4^School of Pharmacy, Rutgers University, New York, NY, United States; ^5^Centre for Inflammation Research, University of Edinburgh, Edinburgh, United Kingdom; ^6^Pathobiology and Diagnostic Investigation, College of Veterinary Medicine, Michigan State University, East Lansing, MI, United States; ^7^AnaPath Services GmbH, Liestal, Switzerland; ^8^Department of Environmental Medicine, University of Rochester, Rochester, NY, United States

**Keywords:** inflammation, cancer, particles, genomic instability, macrophages, neutrophils, hazard assessment

## Abstract

Inflammation is considered a key event in the pathology of many chronic diseases, including pulmonary and systemic particle induced effects. In addition, inflammation is now considered as the key response in standard setting for poorly-soluble low toxicity (PSLT) particles and also the critical endpoint to screen for in OECD based sub-chronic animal inhalation testing protocols. During Particles & Health 2021, an afternoon session was dedicated to the subject and a brief summary of the most important messages are summarized in this paper. In the first part of this session, two speakers (Prof. Lison and Dr Duffin) provided state of the art insight into different aspects and sequels to (persistent) inflammation as a protective or adverse response. Most recent insights on the role of different macrophage cell types were presented as well as perspectives and data provided by inflammatory pathways in humans, such as in asthma and COPD. A brief review of the expert workshop on PSLT particles focusing on the regulatory impact of using persistent inflammation as a key outcome was provided by Kevin Driscoll. The second part of the session focused on the outcomes that are associated with inflammation in animal studies, with an emphasis by Drs. Harkema (Michigan State) and Weber (Anapath) on cell proliferation and other pathologies that need to be considered when comparing human and animal responses, such as outcomes from 14- or 28 day inhalation studies used for specific target organ toxicity classification.

## Novel Insights into Inflammation and Cancer

**Figure 1:**
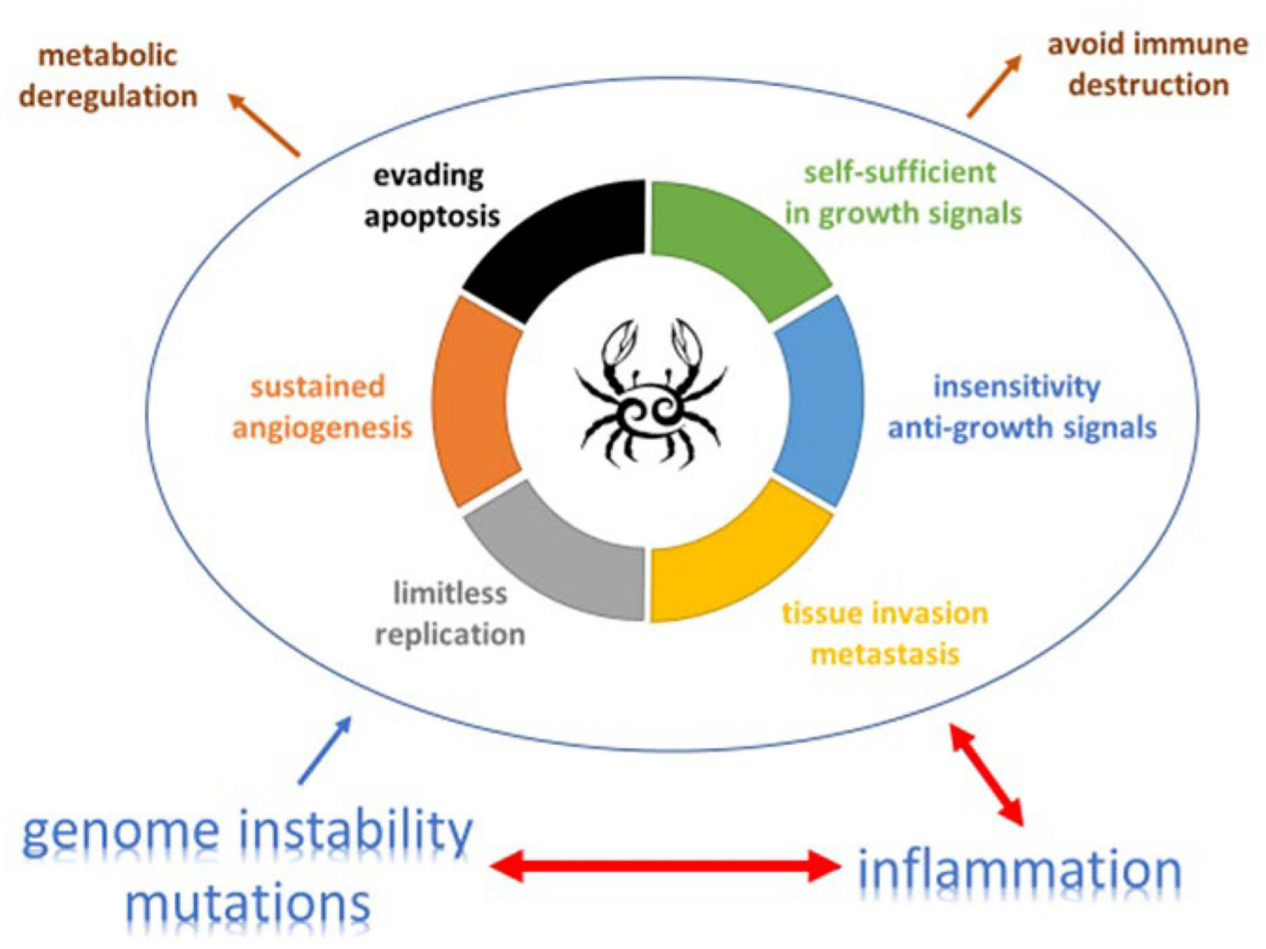
The complex relation and balance between genomic instability, inflammation and cancer promotion and control [adapted from (19) and (20)]. An increasing body of research suggests that two additional hallmarks of cancer are involved in the pathogenesis of some and perhaps all cancers. One involves the capability to modify, or reprogram, cellular metabolism in order to most effectively support neoplastic proliferation. The second allows cancer cells to evade immunological destruction, in particular by T and B lymphocytes, macrophages, and natural killer cells. Additionally, two consequential characteristics (lower half of figure) of neoplasia facilitate acquisition of both core and emerging hallmarks. Genomic instability endows cancer cells with genetic alterations that drive tumor progression. Inflammation by innate immune cells designed to fight infections and heal wounds can instead result in their inadvertent support of multiple hallmark capabilities, thereby manifesting the now widely appreciated tumor-promoting consequences of inflammatory responses.

Prof. Lison (Université catholique de Louvain, Brussels) gave a key note presentation entitled “Macrophages, inflammation and cancer” and presented the paradigm of cancer development proposed by Mantovani et al. (1), which connects inflammation, oncogene activation and genomic instability. The connection between inflammation and cancer includes two pathways: an extrinsic pathway, driven by inflammatory conditions that increase cancer risk (such as in silicosis) and an intrinsic pathway, driven by mutations in cancer cells (such as oncogene activation) which cause inflammation and neoplasia (Figure 1). The intrinsic pathway was uncovered when investigating why inflammatory cells and mediators are present in the microenvironment of most, if not all, tumors. Oncogenes can, for instance, encode tyrosine kinases that are persistently activated in a ligand-independent manner as a result of mutation or chromosomal rearrangement NF-κB is a key transcription factor that coordinates innate immunity and inflammation, but also emerged as an important endogenous tumor promoter (2). The expression of cytokines such as TNF-alpha, TGF-b, IL-6 and other inflammatory mediators is also regulated by NF- κB. NF-κB is crucial both in the context of neoplasia and in the context of inflammatory cell function. It acts downstream of the sensing of microorganisms or tissue damage via the Toll-like receptor (TLR)–MyD88 signaling pathways.

However, one has to realize that inflammation is a dynamic process involving many different effector cells, and these cell populations interact. Macrophages are key in orchestrating the lung inflammatory response to inhaled particles. Recent research has revealed a significant phenotypic heterogeneity in the population of macrophages in lung, and this information has been, so far, insufficiently integrated in particle toxicology. In the lung, as in most other organs, the macrophage phenotype is determined by different parameters, including their ontogeny and tissue localization (niche). Tissue-resident alveolar macrophages (AM) migrated from the embryonic yolk sac and liver to the lung, and have self-renewing potential through local proliferation. Blood monocyte-derived macrophages migrate to the lung in response to insults (viral infection, particle deposition, smoking, etc.,) and can differentiate into interstitial macrophages (IM) or AM. This distinction is important because monocyte-derived macrophages appear to be more reactive than resident AM (3). Following successive insults to which the lung is submitted during life, the population of lung macrophages is thus progressively enriched in monocyte-derived cells, which contributes to increasing sensitivity of the lung to subsequent insults (4). It has also been recently discovered that macrophages associated with blood vessels or nerves in the lung tissue express a specific phenotype. For instance, blood vessel-associated IM appear to exert anti-inflammatory and anti-fibrotic functions (5). This phenotypic heterogeneity has important implications for the selection of appropriate *in vivo* models to evaluate inflammatory response to inhaled particles. For instance, rats that are housed in a clean environment have a high proportion of resident AM, which is totally different from those that are pre-exposed to pathogens such as in real-life conditions. Regarding *in vitro* lung macrophage models used in particle toxicology, the relevance of primary cells (generally obtained from clean animals) and cell lines should be carefully considered in terms of the *in vivo* responses that are being modeled.

Most studies of the mechanisms of cancer-related inflammation have focused on the early stages of pathology, but inflammatory mediators and cells are also involved in the migration, invasion and metastasis of malignant cells. Chemokines and cytokines coordinate autocrine and paracrine interactions between malignant cells and infiltrating leukocytes. These interactions increase the migration, invasion and survival of malignant cells. They also affect the growth of the primary tumor and the ability of tumor cells to colonize the metastatic niche (6).

Then of course there is the inhibitory side of inflammation to tumor growth. There is strong evidence from genetic studies using mouse models that cells of the adaptive immune system carry out surveillance and can eliminate nascent tumors, a process called 3mmune-editing (7). Innate immune responses, which manifest as inflammation, are crucial for the initiation of adaptive immune responses. Therefore, the seemingly divergent effects of inflammation and 3mmune-editing are paradoxical. Yet, a recent study in mice (8) shows that the TLR adaptor MyD88 (which is involved in innate immune responses) has a key role in promoting tumor development and that inflammation-induced carcinogenesis and 3mmune-editing can occur in the same tumor model.

## Resolution of Inflammation as Key to Persistent Effects

**Figure 2:**
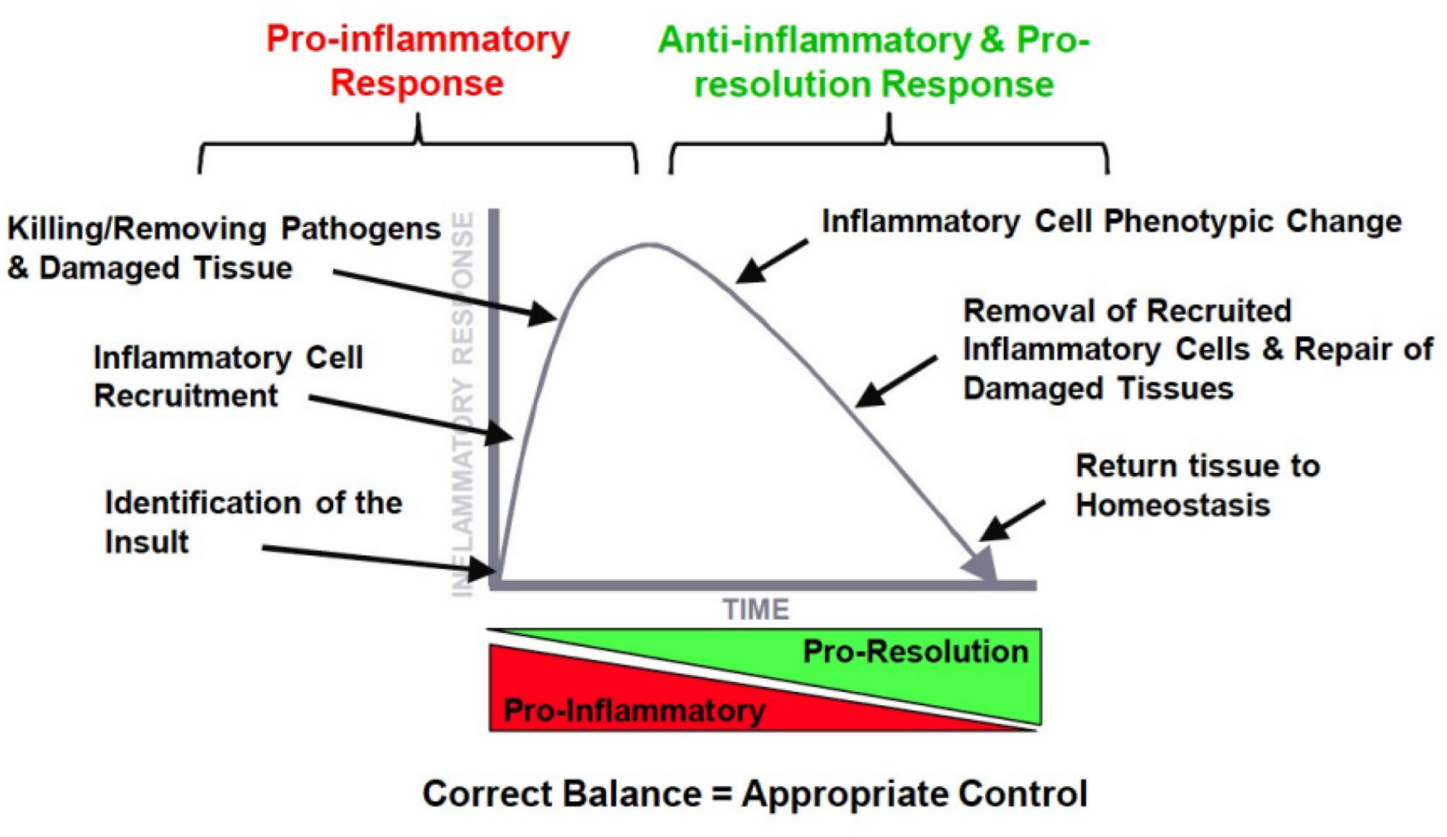
The ideal orchestration of the inflammatory response with an initial pro-inflammatory response and resolution of this response by inflammatory mediators.

Dr. Duffin went into more detail on the time course of events, mediators, and cells during the different phases of the inflammatory response. In Figure 2, it is illustrated how ideally a pro-inflammatory response turns into a second phase after the insult has been eliminated, that is the resolution of the inflammatory profile by an anti-inflammatory network. The ultimate goal is resolution with a return to tissue homeostasis, but, of course, this is different when returning from a neutrophilic inflammation which is designed to kill, or from a macrophage influx which is meant to clear invaders from the lung. Inflammation is essential for normal host defense, but a timely switch from inflammation to resolution is essential to limit host damage. The therapeutic potential for manipulating this process is huge.

Neutrophil apoptosis is a key process in inflammation resolution and can lead to different outcomes such as necrosis, NETosis and necroptosis (9). Especially the formation of NETs (Neutrophil Extracellular Traps) is relatively unknown as they are similar to a conserved anti-microbial defense mechanism (10), releasing DNA complexed to neutrophil proteins. The formation and release of NETs are implicated in tissue damage and inflammation in a number of inflammatory and autoimmune conditions (11) and are highly dynamic processes.

Regarding its meaning for PSLT particles, Dr. Duffin concludes that inflammation is a great marker of particle exposure, but inflammation isn't always a straightforward response. Just as explained by Prof. Lison, inflammation is highly dynamic, highly orchestrated, and at times complex, i.e., acute vs. chronic, innate vs. adaptive response. Timing seems to be key, and we don't seem to know what the difference in impact is between an inflammatory response in an already diseased system and as a primary defense compared to that in a healthy uncompromised organism.

## Inflammation and Overload are Key in Particle Classification

Dr. Driscoll summarized the premises and outcomes of the PSLT workshop in Edinburgh which was held in April 2019 and its outcomes published in 2020 (12). The background is that rats exposed chronically to high concentrations of respirable PSLT materials develop lung cancer (e.g., titanium dioxide, carbon black). However, lung cancer only occurs at doses which overload lung macrophage particle clearance. Moreover:

- Lung cancer is preceded by marked inflammation and epithelial proliferation.
- Lung cancer after PSLT particulate exposure has not been demonstrated in mice and hamsters.
- PSLT materials are not directly genotoxic.

Therefore, lung cancer in the rat is believed to be a consequence of persistent inflammation and epithelial cell proliferation. Until now epidemiological studies in workers with carbon black, talc, or titanium dioxide have not demonstrated associations between PSLT particle exposure and lung cancer in humans. It is interesting, however, to note differences in how regulatory bodies have dealt with observations in a different manner:

- IARC has classified titanium dioxide and carbon black as possibly carcinogenic to humans based on the rat lung cancer data (and absence of increased lung cancer in epidemiology studies). Talc is not classifiable as a human carcinogen (inhalation route).
- The Committee for Risk Assessment (RAC) from the European Chemical Agency (ECHA) classified titanium dioxide as suspected of causing lung cancer through the inhalation route based on a rat data with titanium dioxide and supporting evidence from rat studies with other PSLTs, i.e., carbon black. Carbon black and talc are currently under review based on submissions of France and Netherlands, and expected to follow the same pathway (Class 2 carcinogen).
- NIOSH differentiated the cancer hazard of titanium dioxide based on particle size and the exposure level in rats causing cancer. Ultrafine size (<0.1 μm diameter) titanium dioxide is considered a potential human carcinogen, while the larger size (>0.1 μm diameter) particles are considered unclassifiable as to human carcinogenicity.

The expert panel that convened in 2019 (12) defined a process for characterizing a material as a PSLT, provided guidance on inhalation study design, and reached consensus on OEL setting: the prevention of lung inflammation should be the driving principle in PSLT particle risk assessment and exposure limit setting. More importantly, consensus was based on the relevance of PSLT particulate exposure and rat lung cancer to humans: (i) the rat lung tumors occurring only under lung overload conditions is not relevant to human lung cancer hazard in the context of non-overload conditions, and (ii) PSLT particles should not be considered as human lung carcinogens based on rat data alone and no additional supporting data (from other species, mechanisms).

Dr. Driscoll's key messages included the fact that we need clarity on what materials are PSLTs and which are not, including the importance of particle size in the definition. Of particular note was his observation regarding coal dust, which has been referenced by regulatory agencies as evidence that PSLT particles can overload humans lungs and potentially cause cancer. The issue that Dr. Driscoll highlights is that coal dust particulates are not a PSLT and, so, comparison to titanium dioxide or carbon black does not have a scientific basis. In current regulatory context, substances are classified and missing pieces of evidence are usually taken from other dossiers, such as in TiO_2_. In addition, the current classification in Europe is based solely on lung cancer in rats under conditions of marked overload. Therefore, the PSLT particle hazard classifications need to be revisited to determine if they remain appropriate.

### Pulmonary Cell Proliferation: The Missing Link?

Dr. Harkema's talk focused not on inflammation, but on one of the sequels of inflammation leading to lung remodeling, namely epithelial cell proliferation in toxicant-induced lung injury, repair, adaptation, and cancer. He started off with a seminal paper by Evans et al. (13) and more recently by Aspal and Zemans (14) documenting the signaling pathways involved in this differentiation process. After a brief review of general mechanisms in carcinogenicity, dr Harkema showed how cell proliferation should be considered as a key process in particle induced carcinogenesis (15).

Using data from earlier carbon black inhalation studies he illustrated the development of particle-induced proliferative and inflammatory lesions in rodent lungs. Whereas, initially only inflammation is visible, proliferation is evident after 3 months of exposure, while hyperplasia and fibrosis started to occur at 6–12 months. It takes a lifetime exposure (up to 24 months) in rats to develop lung tumors. However, sustained pulmonary epithelial hyperplasia, a key and necessary factor in carcinogenesis, occurs within weeks to months of particle exposure. Therefore, he went into more detail on the potential use of detecting sustained epithelial cell proliferation in short-term bioassays for predicting particle-induced lung cancer.

The standard process for evaluating substances for carcinogenic activity is to perform a dose range finding study, usually 90 days, in the test species, followed by a 2-year bioassay. Now, short-term (28, 90 days) screening assays in rats and other rodents focus on mode of action and human relevance. However, what is ultimately needed is a determination as to whether the inhaled agent or particle poses a cancer risk to humans and not an investigation to determine if a chemical poses a cancer hazard in rats or mice. He proposed a protocol for a short-term inhalation study with evaluations at 1, 4, and 13 weeks with (1). Postexposure evaluation(s) for sustained proliferation, (2). Histopathologic evaluation of alveolar epithelial hyperplasia and immunohistochemistry for DNA synthesis (e.g., BrdU, PCNA), 3) Morphometric analyses for quantitative pathology (labeling index using digital image analysis), 4) Detecting cell proliferation then developing adverse outcome pathway to evaluate human relevance, 5) Relatively new to the field: incorporate transcriptomic analyses (RT-PCR; bulk tissue RNA sequencing), and 6) Additional analyses such as single-cell RNA sequencing and organoid assays as an *in vitro* target system (16).

### Pathology to Be Reconsidered?

Dr. Weber (Anapath) discussed a number of important issues in relation to current papers on particle toxicology. Study design weaknesses such as small numbers of animals per treatment group, lack of awareness regarding context, and variables that influence test models are not given due consideration. Such papers present bias and also have regulatory consequences. As an example, he discussed a study by Reuzel et al. (17) in which lung fibrosis was noted in rats, but only at a high concentration of synthetic amorphous silicas (SAS). This paper has been crucial in the regulation of amorphous silicas, but, upon independent review of the slides and original materials, a Pathology Working Group comprised of well-recognized pathologists in the field of inhalation pathology did not find fibrosis at all in a single group or animal. In addition, no irreversible chronic persistent inflammation was observed. No substance induced fibrosis, i.e., all SAS types showed comparable effects, although minimal variations in the degree of inflammatory changes were observed and could be related to physicochemical differences (surface area, particle number). The NOAEL of SAS was between 17 and 46 mg/m3 and fibrogenesis was a reversible effect that was observed in all SAS exposure groups. It is recommended that material from future and current studies is kept for independent evaluation since:

- 30 Year-old slides can be de-cover-slipped, re-stained (with standard hematoxylin and eosin staining) and then cover-slipped again, whereby the de-cover slipping may potentially have damaged the original tissue samples and fibrosis might be no longer evaluable;
- Single incidences of minimal focal fibrosis can be observed and fibrosis can be a reversible process. Weber et al. (18) noted such lesions without relation to the concentration and a slight increase in fibrogenesis in the high dose males (2/10). There was also an increase in inflammation indicators, comparable with the other effects noted by Reuzel et al. (17).

The message Dr. Weber wants to bring across is that it seems that pathology is only used as circumstantial evidence and not as the real gold-standard endpoint. In addition, the quality of investigations and resulting interpretation need to be defined more carefully in order to avoid misclassification of effects. The discussion with RAC suggests that also the process of re-evaluation of existing information is not optimally integrated in the classification process. Weber proposed that all studies that might cause a large-scale impact on established chemicals should undergo independent, professional peer review and expert pathological assessment. This could be costly, but should be performed at the discretion of the product owner considering the economical of its outcome.

## Conclusion

Particle induced chronic lung inflammation is seen as the hallmark related to pulmonary effects such as lung fibrosis and lung cancer. Occupational exposure limits for particles are often based on preventing this intermediate, chronic inflammation. The evidence for this Mode of Action is mainly based on rat studies and in that species a one-to-one relationship between inflammation and cancer is assumed. Most studies of the mechanisms of cancer-related inflammation have focused on the early stages of pathology, but inflammatory mediators and cells are also involved in the migration, invasion and metastasis of malignant cells. In addition it is demonstrated that the resolution of inflammation by an anti-inflammatory network and return to tissue homeostasis is an important step in mitigating potential further tissue damage. Structural cell proliferation as the intermediate, local sequel of inflammation should be considered as a key element of *in-vivo* bioassays. Finally, the gold standard for cancer hazard remains pathological evaluation of lung tissue after long term exposures and follow-up and not the short term bio-assays (28 and 90 days) that are often used for particle materials and tend to focus on inflammatory response. Although there is a wealth of evidence of the link between particle-induced inflammation and cancer in the rat, many unknowns in humans are still present and current data (in humans and other species) suggest no such relation. The need remains for a more sophisticated understanding of inflammation in humans during particle inhalation exposures and its role in overall risk of lung cancer.

## Author Contributions

The article is a summary of individual author's contribution to the session. All authors have written their respective paragraphs, while PB and AE have done the main composition, line, and editing of the article. All authors contributed to the article and approved the submitted version.

## Funding

This work is included in the special issue based on Particles & Health 2021-London conference which was party supported by the International Carbon Black Association.

## Conflict of Interest

PB was employed by Nanoconsult Holding BV. KW was employed by AnaPath Services GmbH. The remaining authors declare that the research was conducted in the absence of any commercial or financial relationships that could be construed as a potential conflict of interest.

## Publisher's Note

All claims expressed in this article are solely those of the authors and do not necessarily represent those of their affiliated organizations, or those of the publisher, the editors and the reviewers. Any product that may be evaluated in this article, or claim that may be made by its manufacturer, is not guaranteed or endorsed by the publisher.

## Abbreviations

AM: Alveolar Macrophages
BrdU: 5-bromo-2'-deoxyuridine (incorporation in DNA)
ECHA: European Chemical Agency
IARC: International Agency on Research of Cancer
Il-6: Interleukin 6
MyD88: Myeloid differentiation primary response 88 protein
NETs: Neutrophil Extracellular Traps
NIOSH: National Institute of Occupational Health & Safety
NFkB: Nuclear factor kappa B
NOAEL: No observed Adverse Effect Level
PCNA: Proliferating cell nuclear antigen
PSLT: Poorly Soluble Low Toxicity particles
OECD: Organization for Economic Co-operation and Developmen
RAC: Risk assessment Committee of ECHA
SAS: Synthetic amorphous Silica's
TLR: Toll-like receptor
TGF-B: Transforming Growth Factor-Beta
TNF: Tumor necrosis Factor.
